# Brain morphometry and cognition in late-onset glutaric aciduria type 1: scoping review and novel insights from a case report

**DOI:** 10.1007/s10072-026-08886-9

**Published:** 2026-03-04

**Authors:** Laura Veronelli, Anna Commone, Mara Botti, Paolo Remida, Eleonora Grande, Giuseppe Vallar, Rossella Parini

**Affiliations:** 1 Department of Psychology, University of Milano-Bicocca and Milan Center for Neuroscience, Milan, Italy; 2 Department of Neurorehabilitation Sciences, Casa di Cura IGEA, Milan, Italy; 3 Rare Diseases Unit, Department of Internal Medicine, IRCCS San Gerardo Hospital, via Pergolesi 33, Monza, 20900 Italy; 4 Department of Neuroradiology, IRCCS San Gerardo Hospital, Monza, Italy; 5 IRCCS Istituto Neurologico Mediterraneo Neuromed, Pozzilli, IS Italy; 6 San Raffaele Telethon Institute for Gene Therapy (SR-TIGET), IRCCS San Raffaele Hospital, Milano, Italy

**Keywords:** Glutaric aciduria type 1, Late-onset, Case report, Neuropsychology, Brain volumes, Cortical thickness

## Abstract

**Introduction:**

Glutaric Aciduria type I (GA1) is a rare autosomal recessive organic aciduria, with typical early-onset presentation, characterized by severe movement disorders with damage to the basal ganglia following an acute encephalopathic crisis. Late-onset (LO), milder forms have rarely been described. In LO patients, severe brain damage is frequently reported, including frontotemporal hypoplasia, brain atrophy, subependymal nodules and leukodystrophy. Cognitive impairment is sometimes described, often without formal psychometric assessment.

**Objectives:**

This scoping review aims to synthesize the neuroradiological and neuropsychological features of all LO GA1 patients reported in the literature to date, divided in undiagnosed LO cases detected through selective screening and LO cases with onset of symptoms after 6 years of age.

**Methods:**

The following databases were used: PubMed, Embase and Medline. The search strategy abides by the PRISMA-ScR guidelines.

**Results:**

Out of the 53 LO patients reviewed, extensive quantitative neuropsychological testing was reported in seven cases, while no brain morphometric analysis was performed.

**Case report:**

CASE REPORT. A novel case of a GA1 34-year-old Chinese woman identified through the neonatal screening of her healthy baby is described. Brain morphometric analysis showed diffused reduced volumes and cortical thinning, involving fronto-temporal areas and, to a lesser extent, the parieto-occipital regions. The neuropsychological assessment highlighted mild difficulties in verbal executive functions (inferential thinking) and phonological short-term memory.

**Conclusion:**

The present review and case report suggest that integrating neuroanatomical and neuropsychological investigations into clinical practice may allow a more refined characterization of GA1 patients, contributing to unveil the complex ethio-pathogenic mechanisms underlying the disease and monitor patients over time.

**Supplementary Information:**

The online version contains supplementary material available at 10.1007/s10072-026-08886-9.

## Introduction

Glutaric Aciduria type I (GA1, OMIM 231670) is a rare, autosomal recessive organic aciduria with an estimated worldwide incidence of 1:90.000–1:120.000 newborns [1]. The phenotypic spectrum of GA1 ranges from the classical presentation in infancy, with the onset of a complex and severe hyper- and/or hypo-kinetic movement disorder following an acute encephalopathic crisis, to the milder forms with onset in late childhood, adolescence and adulthood and, at the very end of the spectrum, to asymptomatic individuals, detected through family screening or newborn screening leading to identification of the disease in the mother.

The most common infantile form presents in the first 6 years of life with acute generalized hypotonia, loss of motor skills, feeding difficulties and sometimes seizures, following any kind of triggering stress (such as acute infectious disease, fasting) evolving to a complex movement disorder, featuring a severe permanent dystonia, ataxia and choreoathetosis, with relative sparing of cognition. These children show white matter abnormalities, fronto-temporal hypoplasia and damage to the basal ganglia, particularly the striatum [2]. Most of these infants have progressive asymptomatic macrocephaly, present at or shortly after birth [1]. Insidious onset forms, developing movement disorders and basal ganglia damage without any acute episode have also been described [3].

A minority of patients, showing symptoms from 6 years of age to adulthood, are defined late-onset (LO). They may present with mild, non-specific symptoms, sometimes worsening with age [4–15]. A few asymptomatic LO individuals have been detected through family screening (FS) [16–18]. Furthermore, because of expanded newborn screening, some asymptomatic, or apparently asymptomatic, mothers, have been recognized with GA1 due to low carnitine in their healthy newborns (these mothers will be defined Newborn Screening mothers, ‘NSm’ from now on, in the text) [12, 19–24].

In LO patients, symptoms include headache, macrocephaly, pyramidal tract involvement, dysarthria, ataxia, nystagmus, oculomotor disorders, movement disorders, tremors, orofacial dyskinesia, peripheral neuropathy, and stroke-like episodes. Very few patients were reported with chronic renal disease [25, 26]. The finding of macrocephaly is a strong hint to diagnosis, being frequently present in LO individuals [27]. The differential diagnosis of LO patients includes benign familial macrocephaly and all other causes of macrocephaly, other late-onset forms of organic acidurias, mitochondrial disorders or primary dystonic disorders with onset in adulthood [4, 7, 15, 27]. The choice about the diagnostic pathway of each patient is based first on the family and subject history and clinical examination. The confirmation of diagnosis comes from urine organic acid and molecular analysis [27]. A generic suspicion of genetic disease might also prompt a whole exome sequencing to identify the disease [26]. Recommendations for diagnosis and managing individuals with GA1 have been revised by Boy and colleagues [1].

Mild cognitive delay (in children) and dementia (in adults) are sometimes described [2, 7, 9, 10, 12, 20, 28], but most patients have been reported with preserved cognitive functions [4, 6, 7, 11–15, 17, 19]. However, in most patients no psychometric neuropsychological assessment has been administered.

In LO patients, brain CT and MRI scan, when performed, show severe brain damage, featuring frontotemporal hypoplasia, brain atrophy, subependymal nodules and leukodystrophy in the periventricular and lobar, frontal and parietal white matter, regardless of the severity of the clinical picture [2, 12]. Differently from early-onset GA1, no striatal lesions on cerebral MRI have been found in patients diagnosed in adulthood [1, 5, 7, 12, 15, 29].

Recently, Bian and colleagues [30] investigated grey matter volumes and cortical thickness in a group of GA1 early-onset patients, demonstrating a bilateral reduction of volumes of the basal ganglia, the thalamus, the limbic system, and the right cerebellum, and a bilateral reduction of cortical thickness in the insula, the lateral occipital cortex, the right inferior parietal lobule, the inferior temporal gyrus, and the posterior cingulate cortex, as compared to healthy control participants. In another study, the same group demonstrated that diffusional kurtosis imaging metrics of the temporal lobe and basal ganglia were significantly correlated with dystonia in GA1 children [31].

The objective of this scoping review was to synthesize the clinical, neuroradiological and neuropsychological features of all LO GA1 patients reported in the literature to date, divided in (1) apparently asymptomatic, undiagnosed LO cases detected through FS, or through newborn screening of the healthy baby of a NSm, and (2) LO cases showing evident symptoms after 6 years of age.

Then, we described an allegedly asymptomatic 34-year-old NSm, diagnosed with GA1 through biochemical and genetic tests after the birth of her second child. A brain MRI was performed and findings analyzed, measuring brain structure volumes and cortical thickness. Furthermore, a longitudinal comparison with a brain MRI performed elsewhere 10 years before the present study, allowed to assess the temporal evolution of the brain involvement, in terms of hypotrophy, white matter lesions and subependymal nodules. Finally, the patient’s cognitive profile was assessed through an extensive neuropsychological test battery.

## Review of the literature

### Materials and methods

#### The purpose of review

This scoping review aims to synthesize all LO GA1 described cases in the literature, divided into asymptomatic (FS and NSm), and symptomatic after 6 years of age, with a particular interest on neuroimaging and neuropsychological assessments of these individuals.

This review included all articles in peer-reviewed journals, published in English. Only studies that focused on LO (onset after 6 years of age) GA1 were included. The scope of this review was limited to LO GA1 that was the object of our analysis.

#### Inclusion and exclusion criteria

This review included all articles in peer-reviewed journals, published in English. Onlystudies that focused on LO (onset after 6 years of age) GA1 were included. The scope ofthis review was limited to LO GA1 that was the object of our analysis.

#### Search methodology

A comprehensive electronic search was performed on PubMed, Embase and Medline up to January 2026. The Preferred Reporting Items for Systematic Reviews and Meta-Analyses Extension for Scoping Reviews (PRISMA-ScR) [32] guidelines were applied.

#### Search strategy

The literature search was conducted in January 2026. The initial search focused on identifying articles that contained the following keywords and subject headings in the title and abstract: “glutaric acidemia type I” OR “glutaric aciduria type I” OR “glutaric acidemia type 1” OR “glutaric aciduria type 1” OR “glutaryl-CoA dehydrogenase deficiency” AND “late onset”. Subsequently, we performed a second search across the databases for all articles containing the following keywords and subject headings: “glutaric acidemia type I” OR “glutaric aciduria type I” OR “glutaric acidemia type 1” OR “glutaric aciduria type 1” OR “glutaryl-CoA dehydrogenase deficiency” AND “case report”. Finally, we searched the reference lists of the identified full text articles for additional sources that met the established inclusion criteria.

#### Data extraction

The reviewers used a data extraction form to record information about each patient identified. The form contained information about the presence of macrocephaly, symptoms, genetic information, MRI and neuropsychological summary, treatment and outcome. A summary of the key information about each patient is shown in Tables 1 and 2.

#### Study screening and selection

Duplicates, conference abstracts, papers written in languages different from English and papers about different diseases were consecutively removed. Two independent reviewers (RP) and (LV) evaluated the eligibility of the identified articles using inclusion and exclusion criteria. A third reviewer was selected to resolve any conflicts during the search process (MB).

### Results

**Fig. 1:**
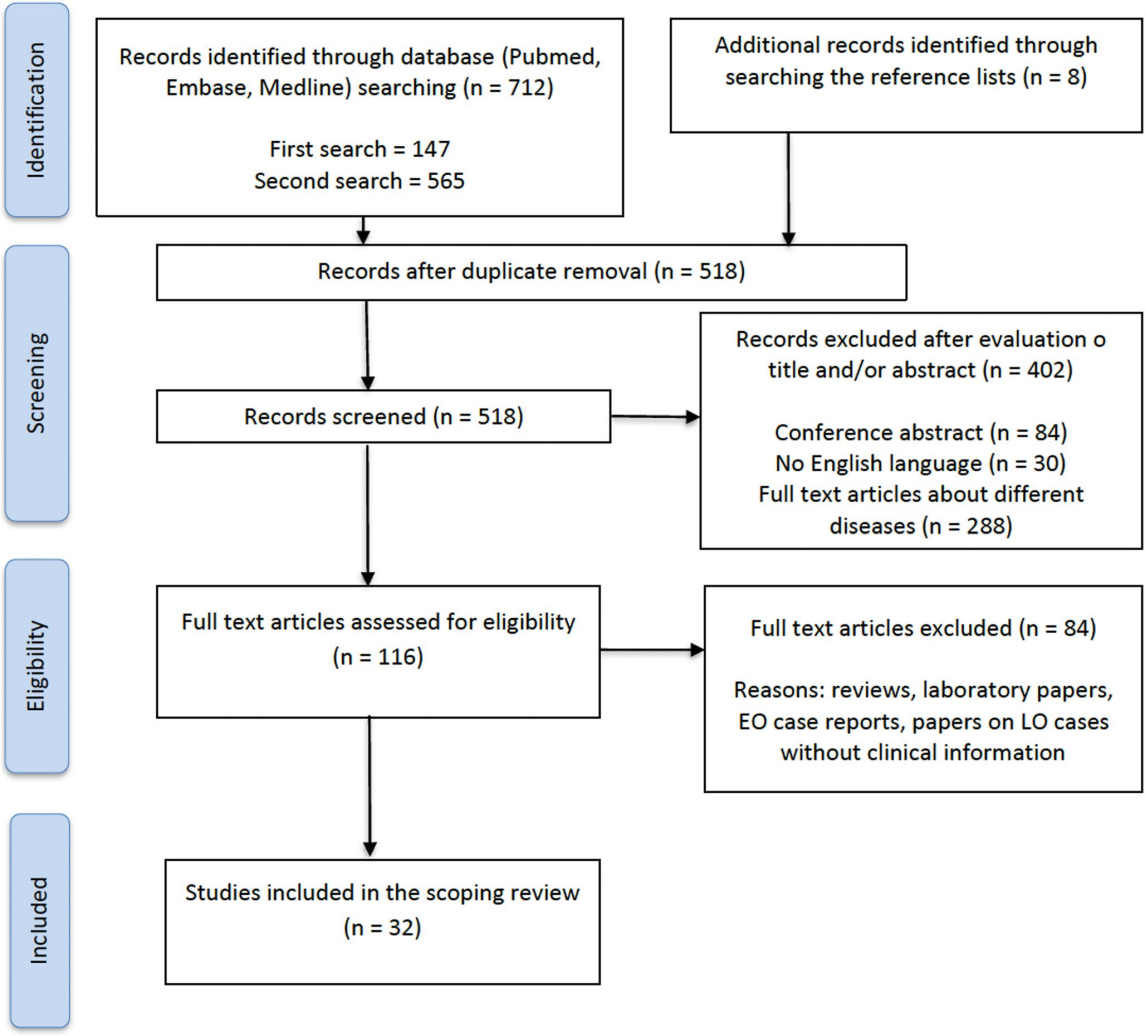
PRISMA flowchart illustrating the search process

The search yielded 712 articles through database searching, and 8 records were identified through searching the reference lists. After the review process, a total of 32 articles were found to be relevant to our topic of interest. A detailed schema of the results of the search process can be found in Fig. 1. The total number of identified LO patients in this review was 53: 24 were LO apparently asymptomatic individuals, identified through FS or NSm (see Table 1, the first line reports data about the additional patient described in the present paper), and 29 were LO patients identified through reported symptoms (Table 2). In general, mention of brain MRI/CT data was found for 42 patients. Quantitative neuropsychological assessments were reported for seven patients, while other three patients underwent a screening test (Mini Mental State Examination; MMSE).

As shown in Table 1, out of the 24 asymptomatic patients reported in the literature (mean age = 22.2 ys; median age = 24 ys) 11 were identified through FS and 13 were NSm. Macrocephaly was present in eight patients, not present in other eight and for the remaining eight individuals the information was missing. Six out of 13 affected mothers had had mild symptoms of the disease before the diagnosis, mainly headache and fatigue [19, 24]. One patient had hypotonia and learning disabilities [20] and another one had developed coordination deficits and cognitive disabilities after diagnosis [12]. The eleven subjects recognized through FS had been completely asymptomatic before diagnosis.

In 14 of the 15 patients who underwent brain MRI, fronto-temporal hypoplasia, white matter abnormalities and subependymal nodules were reported, while only one individual, identified by FS, had normal MRI [17]. No quantitative measurements of brain volumes or cortical thickness have been reported. No changes of neuroradiological findings were found after treatment.

Only three patients (cases n. 3, 6 and 9 in Table 1) underwent a quantitative neuropsychological evaluation, limited to the Wechsler battery, detecting normal cognition (case 3, FS [33]), and mild (case 6, FS [34]), and moderate cognitive deficit (case 9, NSm [20]). Mitigation of symptoms is frequently reported after short-time L-carnitine treatment, but there are also patients exhibiting new signs and symptoms, years after starting L-carnitine treatment [12]. The long-term outcome of the few asymptomatic patients identified through FS [16, 17] is known only for three patients (case n. 2, 4, 10) reported respectively by Gürbüz and colleagues [35], and by Stepien and colleagues [18].

Table 2 summarizes LO patients detected through symptoms (29 cases). Their ages at diagnosis varied from 8 to 71 years (mean age = 31.3 ys; median age = 34.5 ys). Macrocephaly was reported in nine individuals and not present in five. This information was missing for the other 15 patients. Onset was always insidious, mainly with mild symptoms, most frequently headache. Neuroradiological features were similar, independent of symptoms’ severity, with no report of volumetric and cortical thickness measurements. Three LO patients detected through symptoms (case n. 12, 26, 28) were assessed using only the MMSE [5, 7, 9], two of whom had mild [9] and severe cognitive impairment [7], while the other obtained a score within normal range [5]. Two patients (case n. 2 and 23 [2, 4], underwent the Wechsler scales (average range). Only two studies (case 9 and 18) reported an extensive neuropsychological assessment [6, 13], the former describing delayed psychomotor development in a 16 years-old patient (WAIS 92: verbal 95, performance 99), with slight difficulties in attention, execution and fine motor function; the latter reporting a cognitive profile within normal range, with the exception of mild stress related anxiety and depression, in a 35 years-old Turkish Cypriot female.

**Table 1:** Asymptomatic or undiagnosed LO cases detected through neonatal or family screening. The case described in this paper is the first of the list, with no number

	Sex (F/M), Age (ys), FS/NSm, [ref]	Macrocephaly yes/no	Symptoms	Genetic variants	MRI summary	NPSY evaluation	Treatment	Outcome
	F, 34, NSm (present case)	Yes	Headache; episode of hypotonia and walking impairment in the past during infection	c.1244-2A>C/ c.1244-2A>C	Fronto-temporal hypoplasia and hypotrophy, leukoencephalopathy in anterior (frontal) and paratrigonal regions and subependymal nodules	Mild difficulties in verbal executive (inferential thinking) and phonological short-term memory	L-Carnitine	Headache improved
1	F, 6, FS [17]	No	No	c.1204C>T[p.Arg402Trp]/ c.532G>A[p.Gly178Arg]	Normal	Na	Diet, L-carnitine, riboflavin	Favorable, 2 months follow-up
2	F, 7.25, FS [35]	No	No	c.743C>T[p.Pro248Leu]/ c.1204S>T[p.Arg402Trp]	Typical of GA1	No motor-mental delay	Diet, L-carnitine, riboflavin	Favorable at 22 years
3	M,10, FS [33]	Yes	Slightly delayed language acquisition	Na	Bilateral frontotemporal atrophy and mild ventricular enlargement	WISC-R full-scale IQ=107, verbal IQ=100, performance IQ=115;	Probably not treated	Na
4	F, 11, FS [18]	Na	No	c.553G>A[p.Gly185Arg]/ c.1204C>T[p.Arg402Trp]	Mild dilatation of lateral ventricles, widening of the Sylvian fissures and bilateral enlargement of the fluid space anteriorly to the temporal poles	Na	Diet and L-carnitine since 11 years	Favourable, MRI stable after 4 years
5	M, 11, FS [16]	No	No	c.395G>A/ c.1172-1173insT	Na	Na	L-carnitine, riboflavin, diet	Na
6	F, 13, FS [34]	Yes	Difficulties in school performance from one month before	c.1204S>T[p.Arg402Trp]/ c.1204S>T[p.Arg402Trp]	Symmetric signal changes of supratentorial white matter (preferentially of frontal areas). Optic radiation spared.	WISC: full-scale IQ=82, verbal IQ=88, performance IQ=80; mild deficits in working memory (Free and Cued Selective Reminding Test) and executive functions (Tower Of London) (test scores not reported)	Diet and L-carnitine	Na
7	M, 17, FS [16]	Yes	No	c.1064G>A/ c.1147C>T	Leukoencephalopathy, enlarged sylvian fissures	Na	L-carnitine, riboflavin, diet	Na
8	F, 22, NSm [12]	No	No	c.383G>A[p.Arg128Gln]/c.383G>A[p.Arg128Gln]*	Frontotemporal hypoplasia, WM changes, dentate nucleus	Na	Diet, L-carnitine	Headache
9	F, 24, NSm [20]	Yes	Hypotonia, learning disabilities	[p.Arg402Trp]/ [p.Gly390Val]	Typical features of GA1	IQ 75 (WAIS)	Na	Na
10$	M, 24, FS [36]	Na	Intermittent fatigue	c.1060G>A[p.Gly354Ser]/c.1154C>T	Na	Na	L-carnitine	Fatigue improved
11	F, 25, NSm [12]	Yes	No	[p.Glu365Lys]/ [p.Glu365Lys]	Frontotemporal hypoplasia, WM changes, dentate nucleus	Na	No	Headache, intermittent vertigo
12	F, 28, NSm [24]	No	Intermittent headache, dizziness, forgetfulness	c.641C>T[p.Thr214Met]/ c.1204C>T[p.Arg402Trp]	Bilateral opercular atrophy, subcortical and periventricular WM involvement, symmetrical hyperintensities in the putamen	Na	L-carnitine, Diet	Na
13	F, 29, NSm [12]	No	No	[p.Arg128Gln]/ [p.Arg402Trp]	Frontotemporal hypoplasia, WM changes	Na	Diet, L-carnitine	Headache, coordination deficits, cognitive disability
14	M, 30, FS [37]	No	No	c.877G>A[p.Ala293Thr]/ [p.Thr193_Arg194ins His]	Subependymal nodules	Borderline IQ	Na	mild
15	F, 31, NSm [23]	Na	Na	c.1063C>T[p.Arg355Cys]/ c.769C>T[p.Arg257Trp]	Na	Na	Na	Na
16	F, 32, NSm [23]	Na	Na	c.1244-2A>C/ c.1244-2A>C	Na	Na	Na	Na
17	F, 33, NSm [24]	Yes	Chronic fatigue, vertigo	c.263G>A[Arg88His]/ c.263G>A[Arg88His]	Subcortical enlargement in the frontal part of both cerebral hemispheres, symmetrical hyperintensity in subcortical area, periventricular region and corpus callosum	Na	L-carnitine	Na
18$	M, 33, FS [36]	Yes	Learning difficulties in childhood. NO symptoms at present	c.1060G>A[p.Gly354Ser]/c.1154C>T	Two arachnoidal cysts at CT scan	No cognitive complaints, works as a mechanic	Na	Na
19	F, 35, NSm [38]	Na	No	c.1204C>T[p.Arg402Trp] / c.1262C>T [p.Ala421Val]	Na	Na	L-carnitine	Na
20$	F, 35, NSm [36]	Na	Two fainting in childhood, difficulties in performing sports, From 30yo onwards complaining of severe fatigue	c.1060G>A[p.Gly354Ser]/c.1154C>T	Enlarged sylvian fissures	No intellectual problems or movement disorders	Diet, L- carnitine	Fatigue improved
21§	M, 41, FS [35]	Yes	No	c.1093G>A [p.Glu365Lys]/ c.1093G>A[p.Glu365Lys]	Na	No motor-mental delay	-	Na
22	F, nr, NSm [22]	No	No	c.1204C>T[p.Arg402Trp]/ c.853-26_854del	Na	Na	Diet, L-carnitine	Na
23	F, nr, NSm [19]	Na	Intermittent fatigue	c.11delG/ c.682T>G[p.Cys228Phe]	Na	Na	L-carnitine 330mg/kg/BID	Mitigation of symptom
24	F, nr, NSm [19]	Na	Intermittent fatigue	c.542A>G[Glu181Gly]/ c.542A>G[Glu181Gly] (or hemizygous?)	Na	Na	L-carnitine 330mg/kg/BID	Mitigation of symptom

Neonatal (NSm - female mothers) or family screening (FS); *WM *white matter; *Na *not available. Molecular data are reported, as variants at c.DNA (c.) and protein (p.) levels when both available. * in the original paper this variant is reported, probably by mistake, as p.Pro128Gln. § Patient 21 is uncle of patient 5 Table 2. $ Patients 10, 18, 20 are two brothers and a sister of the same family; index case pt. 20 mother identified through NS of her healthy baby

**Table 2:** LO cases diagnosed by symptoms after 6 years of age, listed by age at diagnosis

	Sex (F/M), age at diagnosis (ys) [ref]	Macrocephaly yes/no	Symptoms	Genetic variants	MRI summary	NPSY evaluation	Treatment	Outcome
1	F, 8 [16]	Yes	Transient regression, seizure, headache, stagger during infection	c.1064G>A/ c.1147C>T	Leukoencephalopathy	Na	Diet, L-carnitine	Na
2	F, 8.5 [2, 12]	No	Nausea, vomiting, headache, ataxia	[p.Arg128Gln]/ [p.Glu414Lys]	Extensive WM abnormalities in serial MRI from 8.5 to 15.7 ys	IQ 96 (Hamburg Wechsler Intelligence Test for children, third edition)	Diet, L-carnitine	Improved, no symptoms at 17 years, MRI unchanged
3	M, 9 [16]	Yes	Headache, stagger	c.411C>G/ c.979G>A	Enlarged sylvian fissures, leukoencephalopathy	Na		
4	F, 12 [17]	Na	Intermittent headache	c.1204C>T[p.Arg402Trp]/ c.532G>A[p.Gly178Arg]	WM degeneration, bilateral temporal lobe arachnoid cysts	Normal intelligence	Diet, riboflavin and L-carnitine	No changes after two months
5§	F, 12 [35]	Yes	Mild motor-mental retardation	c.1093G>A [p.Glu365Lys]/ c.1093G>A[p.Glu365Lys	Typical	Mild motor-mental delay	Diet, riboflavin and L-carnitine	-
6	M, 14.6 [2]	Na		[p.Arg88Cys]/ [p.Arg88Cys]	Nr	No disabilities	Diet, L-carnitine	Na
7	M, 15 [7, 12]	Yes	Vertigo, headache, ↓ motor balance and fine motor skills	[p.Arg88Cys]/ [p.Arg88Cys]	Mild frontotemporal atrophy, hypoplasia, supratentorial WM hyperintensity mainly around frontal and temporal lobes	Mild delay, slightly reduced motor skills	Diet, L-carnitine	Complete recovery
8	F, 16 [10]	Yes	Fainting after exercise, mild cognitive delay	c.1204C>T[p.Arg402Trp]/ c.1244A>G(IVS10-2A>G)	Enlargement of temporopolar CSF spaces, widened Sylvian fissures and mesencephalic cistern, periventricular and deep WM hyperintensity, hyperintensity of splenium of corpus callosum, dentate nucleus and substantia nigra	Mild learning difficulties but she proceeded normally at school	Diet, L-carnitine	No progression of MRI after 1 year
9	F, 16 [6]	No	3-year history of hand tremor	c.877G>A[p.Ala293Thr]/ c.1204C>T[p.Arg402Trp]	High bilateral signal of putamen and diffuse bilateral abnormalities of WM with a major involvement in frontal areas	Delayed psychomotor development. WAIS 92 (verbal 95, performance 99), slight difficulties in attention, execution and fine motor function	L-carnitine	At 19 years she had tremors, dyskinesia and marked nasal voice
10	M, 18 [12]	No	Headache	[p.Tyr74fs]/ [p.Arg132Gly]	Frontotemporal hypoplasia, WM changes, subependymal lesions	Na	Diet, L-arnitine	No symptoms at age 31
11	M, 18 [28]	Na	Age onset not reported. Progressive Leukoencehalopathy, cognitive deficit, neuropsychomotor regression, extrapyramidal symptoms (tremors)	c.91+5G>A(IVS1+5G>A)/ c.262C>T[p.Arg88Cys]	Na	Neuropsychomotor regression	Na	Na
12	F, 19 [5]	No	4-month history of recurrent unspecific Headache, horizontal nystagmus, upward gaze palsy, convergence paralysis, slowing of fine motor function of left hand	c.219delC[p.Tyr74fs/c.394C>G[p.Arg132Gly]	WM abnormalities, no atrophy	MMSE 29 (IQ 94)	Na	Na
13	M, 19 [26]	Na	2 years history of chronic renal disease, 5 month history of weakness proximal and distal lower limbs. Absent tendon reflexes	c.1207C>T[p.His403Tyr]/ c.1207C>T[p.His403Tyr]/	Bilateral symmetrical white matter hyperintensities with diffusion restriction,subependymal nodules and involvement internal capsule	No cognitive or behavioral disorders	Diet, L-carnitine, riboflavin	No weakness improvement after 6 months
14	M, 20 [8]	Na	Recurrent headache, hyperreflexia	Na	Bilateral symmetric periventricular signal intensity changes (leukoencephalopathy), restricted diffusion in DWI	Na	L-carnitine	Frequency and severity of headache declined
15	M, 26 [28]	Na	Age onset not reported. Neurological regression, dystonia	c.1204C>T[p.Arg402Trp]/ c.1204C>T[p.Arg402Trp]	Leukoencephalopathy, putamen lesions	Neuropsychomotor regression	Na	Na
16	F, 29.3 [2]	Na	Neurological	Na	WM signal abnormalities	Na	Diet, L-carnitine	Na
17	F, 32 [39]	Na	Headache and bipolar disorder	c.636-10_642dup[p.Asn215fs]/ c.1093G>A[p.Glu365Lys]	Confluent symmetric bilateral WM signal abnormalities, mild atrophy	Reduced verbal fluency and motor sequencing suggestive of frontal lobe dysfunction (no test scores)	L-carnitine	Improved mood and reduced headache after 3 months of treatment
18	F, 35 [13]	Na	Headache and subjective loss of memory	c.1204C>T[p.Arg402Trp]/ c.1204C>T[p.Arg402Trp]	Frontotemporal hypoplasia, periventricular WM changes, bilateral subependymal nodular lesions (some cystic)	Complete neurocognitive testing within normal range. Mild stress related anxiety and depression	Diet, L-carnitine	Na
19	M, 35 [40]	Yes	Persistent headache and blurring vision in the past 3 months	c.183_184delCA[p.Thr62fs*2]/ c.183_184delCA[p.Thr62fs*2]	Leukoencephalopathy. frontotemporal atrophy	Normal neuropsychological assessment (specific tests not reported)	Diet, L-carnitine	Reduced headache episodes after 3 months
20	M, 41 [15]	No	Seizures, nystagmus, hyperreflexia	c.937C>T[p.Arg313Trp]/ c.383G>A[p.Arg128Gly]	Mild frontotemporal hypoplasia, periventricular, subcortical and WM changes, multiple subependymal nodular and cystic lesions	Normal mental status and motor function	Diet, L-carnitine	No seizures or other neurological symptoms after two years
21	F, 45 [14]*	Na	Slight dysarthria, generalized chorea and dystonia.	c.1148G>A[p.Arg383His]/ c.1262C>T[p.Ala421Val]	Very mild bilaterally symmetric gliosis in the dorsal putamen, no significant atrophy.	Na	Diet, L-carnitine, riboflavin	Stable 4 years after starting treatment
22	F, 45 [14]*	Na	Dystonia of the neck, mild orofacial dystonia, slight dysarthria, jerky chaotic movements of the arms during rest, posture and action.	c.482G>A[p.Arg161Gly]/ c.1262C>T[p.Ala421Val]	Abnormalities of the dorsal putamen	Na	No dietary treatment, only emergency treatment	After 6 years slight progression of movement disorders
23	F, 50 [4]	Na	Dysarthria, lingual dystonia, orofacial dyskinesia, hypoplastic right leg (she used calipers and two sticks to walk), dystonic posture and clawing of both hands	Na	Cortical atrophy, large CSF spaces around anterior portion of both temporal lobes, WM abnormalities	Verbal IQ 93 (WAIS-R). Good scores for non-verbal reasoning	Na	Na
24	M, 51 [15, 16]	Yes	Paroxysmal and then continuous weakness in legs with speech disturbances, gaze palsy, central facial palsy and hemiparesis on the left side	c.533G>A[p.Gly178Glu]/ c.1205G>A[p.Arg402Gln]	Cerebral infarction, restricted diffusion in right frontal lobe, occipital lobe and basal ganglia, frontotemporal hypoplasia, brain atrophy, several subependymal nodules	Normal cognition	Diet, L-carnitine	After 13 years severe leukoencephalopathy, multiple infarctions in right cerebral hemisphere, occlusion of left intracranial vertebral artery
25	F, 55 [11]	Na	6-year history of episodic lower extremities numbness and paresthesias	c.1219C>G[p.Leu407Val]/ c.848delT[p.Leu283fs]	WM abnormalities, multiple subependymal nodules, temporal lobe hypoplasia, mildly generalized cortical atrophy	Na	Na	Na
26	M, 56 [9]	Na	Feet pain, leg weakness, speech disturbance and incontinence since 30 years. At examination proximal and distal weakness of both legs with diminished reflexes, normal tendon reflexes in the arms, pes cavus. Nerve conduction studies showed peripheral neuropathy	c.301G>A[p.Gly101Arg]/ c.301G>A[p.Gly101Arg]	Communicating hydrocephalus, bilateral frontotemporal atrophy and temporal arachnoid cysts, prominent periventricular and deep leukodystrophy, subependymal cauliflower-like mass lesion	MMSE 25/30. Impaired executive functions, working memory, difficulties in learning new information	Carnitine	Cognitive decline after 3 years, no change in brain MRI
27	M, 65 [2]	Na	Na	[p.Arg383Cys]/ [p.Arg383Cys]	WM signal abnormalities	Severe	Diet, L-carnitine	Slight improvement
28	M, 66 [7, 12]	Yes	Headache ≥ 35 years tremor ≥ 50 year seizures ≥ 54 years dementia, ↓ speech ≥ 63 years	[p.Arg383Cys]/ [p.Arg383Cys]	WM changes, frontotemporal hypoplasia, subependymal lesions	Severe mental deficit (MMSE: 8). Partial improvement after 3 months (MMSE: 16)	Diet, L-carnitine	Dementia, tremor, epilepsy, ↓ speech, intermittent orofacial dyskinesia, dysmetria, ↓ fine motor skills
29	M, 71 [28]	Yes	Repetitive cerebral ischemia, intention tremor ≥ 62 years confusion, ↓ memory ≥ 65 years, epilepsy ≥ 72 years	[p.Arg402Trp]/ [p.Ala421Val]	WM changes, subependymal lesions, frontotemporal hypoplasia	Na	Diet, L-carnitine	Progressive dementia, tremor, epilepsy, dysdiadochokinesis, incontinence

*WM *white matter;* Na *not available; * symptoms present in childhood and not diagnosed. Molecular data are reported, as variants at c.DNA (c.) and protein (p.) levels when both available. § Patient 5 is nephew of patient 21 in Table 1

## Case report

The patient was a 34-year-old woman of Chinese origins. Her family history was unremarkable, and she had always been in good health, except for a single episode of diffuse hypotonia, accompanied by walking impairment, during a probable sepsis originated from a urinary tract infection when she was 23 years old. In that occasion the patient was taken to the local hospital and treated in emergency with levofloxacine immediately after performing blood culture, considering her critical conditions. Urine culture was done on the first urine sample after admission. Blood tests showed very high erythrocyte sedimentation rate (79 mm/hr), and C-reactive protein (239 mg/L; nv < 5). White blood cells were15,730/ mm^3^ with neutrophils 88%. Increased leukocyte esterase at dipstick and many white blood cells and red blood cells were found in the urinary sediment. Blood and urine culture resulted negative. Hyposthenia and walking impairment progressively disappeared in about 2 weeks. A brain CT scan performed at that time showed a bilateral anterior temporal cystic hygroma with temporal hypoplasia and diffused cortical hypotrophy; moreover, some subependymal nodules were found in the walls of the lateral ventricles. A subsequent brain MRI, performed on a T1 scanner three weeks after the onset of symptoms, when they had already disappeared, confirmed the findings mentioned above, and highlighted a T2 and FLAIR hyperintensity of the periventricular white matter, with corresponding slight T1 hypointensity and increased ADC. No involvement of the subcortical U-fibers or the striatum was detected. She was then referred to a local neurologist, who, probably on the basis of subependymal nodules, hypothesized a diagnosis of Tuberous Sclerosis complex, suggesting a referral to a highly specialized center, that was not followed through. Subependymal nodules are typically found in Tuberous Sclerosis Complex (TSC). In TSC though they are usually accompanied by other, more characteristic, findings such as the pathognomonic Cortical Tubers [41].

The woman came to our attention when her second son was recalled by the newborn screening team due to low free carnitine levels (C0 = 3.76 μm; cut-off 6.11) at MS/MS Extended Newborn Screening test (see Table 1, first row).

Metabolic tests performed in the woman revealed very low serum free carnitine levels (1.49 umol/L, normal values 25.00–54.00), elevated glutaryl carnitine and the presence of glutaric ad 3-OH- glutaric acids in urine (MS/MS), leading to a diagnosis of GA1, subsequently confirmed by genetic analysis (c.1244–2 A > C in homozygosity and c.1261G > A in heterozygosity).

The neurological examination was normal, notably with no signs of extra-pyramidal involvement. Macrocephaly was detected (59 cm). A second brain MRI was performed on a 1.5 T scanner, 10 years after the first one. The MR imaging protocol included the following sequences: a three-dimensional fluid-attenuated inversion recovery (3D FLAIR), three-dimensional diffusion-weighted imaging with isotropic acquisition (3D DWI iso), axial T2-weighted turbo spin-echo (T2 TSE), fast susceptibility-weighted imaging (SWI-P fast), three-dimensional T1-weighted turbo field echo (3D T1 TFE), and three-dimensional pseudo-continuous arterial spin labeling (3D pCASL) for perfusion assessment. The study confirmed the widespread hyperintensity of the periventricular white matter, with confluent morphology, multiple subependymal nodules, located at the ventricular roof and the septum pellucidum, moderate enlargement of ventricular mid-cells, and marked bilateral fronto-temporal and cerebellar vermian hypoplasia, with enlargement of the CSF spaces. A therapy with L-carnitine was initiated, resulting in a normalization of the free carnitine levels. A monitoring of the patient’s urinary glutaric acid levels confirmed that she was a high excretor.

The patient, with 8 years of education, demonstrated good Italian proficiency (she reported having been in Italy since the age of 8–9 years). Verbal comprehension, assessed through everyday life topics, was good, although she needed clarification for some more complex sentences. The patient, who runs a coffee shop together with her husband, reported using mainly Chinese language at home. From an emotional-behavioral perspective, the patient showed no clinical abnormalities, except for fatuity, and a slightly depressed mood (Beck Depression Inventory-II score = 9/63, cut-off 10 [42]). The patient reported no motor or cognitive difficulties, decline in daily functioning or other symptoms or complaints.

The study was conducted according to the standards of the Declaration of Helsinki, and the patient signed a written informed consent prior to participate. The case report follows the CARE guidelines.

### Materials and methods

#### Neuroanatomical data

Neuroanatomical data were analyzed considering the presence of hypoplasia/hypotrophy, leukoencephalopathy with extra-striatal vs. striatal lesions, and subependymal nodules, as typical features of LO GA [12]. Hypotrophy was quantitatively measured through volumetric and cortical thickness analyses.

##### Brain morphometric analysis.

Structural 1.5T MRI was analyzed to quantify brain volumes and thickness. The T1-weighted structural image (MPRAGE) was processed using vol2Brain (https://volbrain.net/services/vol2Brain [43], an online tool that offers automatic brain segmentation. The vol2Brain processing pipeline involved several steps: denoising through spatially adaptive non-local means; initial inhomogeneity correction via the N4 method; affine registration to the MNI152 space using ANTS software; fine inhomogeneity correction based on SPM; intensity normalization; extraction of the intracranial cavity; tissue classification; brain subregions segmentation; and subcortical structure segmentation.

Vol2Brain produces several binarized nifti masks, both in native and in MNI normalized space, corresponding to segmentations of brain macroscale, i.e., total intracranial volume, gray matter, white matter, CSF, as well as primary subcortical structures (including caudate, putamen, thalamus, globus pallidus, hippocampus, amygdala, and accumbens), and the lateral ventricles and cortical subregions covering the entire brain. The vol2Brain pipeline outputs report summarizes volumetric data for all segmented structures. Additionally, vol2Brain provides population-based normal volumes and left-right asymmetry boundaries, accounting for sex and age, derived from a dataset of 600 neurologically intact subjects spanning most of the adult lifespan [43]. This tool allowed us to perform a thorough investigation of the patient’s brain anatomy.

##### Leukoencephalopathy and subependymal nodules.

 Leukoencephalopathy and subependymal nodules were evaluated through clinical inspection and lesion quantification, using the Fazekas scale score [44] for white matter involvement.

#### Neuropsychological assessment

The Montreal Cognitive Assessment [45] and the Clock Drawing test [46] were administered as screening tests. Furthermore, a deep neuropsychological assessment investigated the following cognitive domains: language, praxis, spatial cognition, logical-deductive abstract reasoning, executive functioning, verbal and visual short-term and long-term memory. The complete list of the tests administered is reported in Supplementary Materials.

### Results

#### Neuroanatomical data

##### Brain morphometric analysis. 

The vol2Brain volumetry report presents brain volumes for cortical subregions, compared to normative values based on age- and sex-matched populations. The volumes and cortical thickness values of the whole brain subregions and expected 95% limits of normalized volume in function of sex and age for each measure are detailed in Tables S1 and S2.

*Volumes*

Notably, in our LO patient we identified different degrees of hypotrophy affecting both the right- and left-hemisphere grey matter volumes (262.20 and 263.87 cm3/%, respectively, without significant left-right side differences: asymmetry − 0.6343). White matter volumes were within normal ranges (right: 261.08 cm3/%; left: 253.56). A similar picture emerged for the cerebellum, with reduced grey matter (right: 43.57; left: 43.36; vermis: 6.32) and normal white matter volumes (right: 17.02; left: 16.95). The Cerebrospinal Fluid (CSF) was much higher than expected (26.059).

**Fig. 2:**
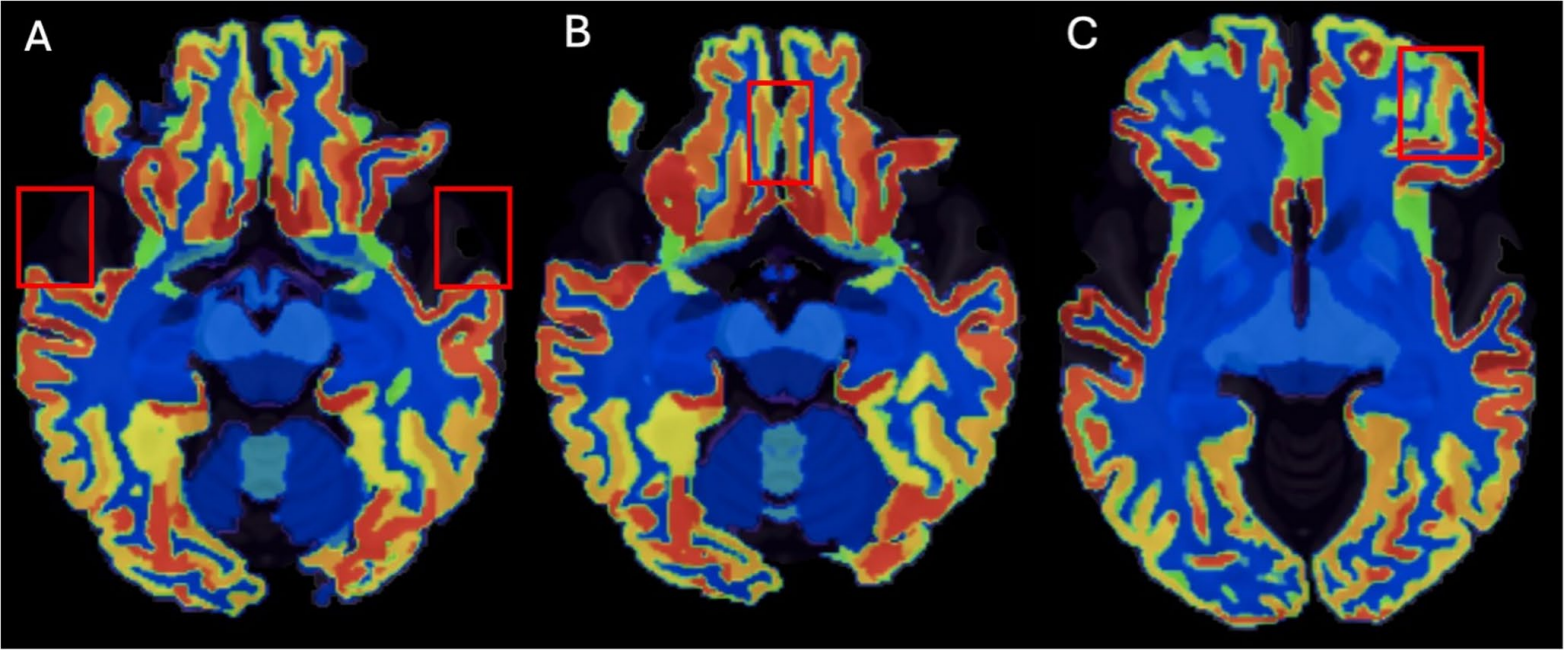
Reduced cortical volumes in the left and right temporal poles (panel A), in the medial frontal cortex (panel B) and left lateral orbitofrontal gyrus (panel C, radiological convention)

Abnormally reduced volumes included the fronto-temporal lobe, as well as parietal and occipital areas. Hypotrophic/hypoplasic regions predominantly included the temporal pole (right: 0.000; left: 0.004), the planum polare (right: 0.064; left: 0.101), the left planum temporale (0.093), the entorhinal area (right: 0.057; left: 0.081), the middle cingulate gyrus (right: 0.208; left: 0.260), the superior temporal gyrus (right: 0.322; left: 0.244), the medial frontal cortex (right: 0.044; left: 0.049), the triangular inferior frontal gyrus (right: 0.185; left: 0.154), the left lateral orbital gyrus (0.047), the supplementary motor cortex (right: 0.252; left: 0.232), the frontal operculum (right: 0.087; left: 0.094), the precuneus (right: 0.560; left: 0.501), and the left supramarginal gyrus (0.406). Figure 2 shows reduced cortical volumes in the left and right temporal poles (panel A), in the medial frontal cortex (panel B) and in the left lateral orbitofrontal gyrus (panel C). Concerning striatal nuclei, a slightly reduced volume was observed for the putamen (right: 0.243; left: 0.242), and not for accubens, caudate nucleus and globus pallidum. Lobules I-V and VI-VII of the cerebellar vermis were reduced (0.130 and 0.085, respectively), and not lobules VIII-X (0.177). Additionally, the right inferior lateral (0.073), lateral (right: 2.022; left: 2.046) and third ventricle (0.119) volumes exceeded the normative range.

*Cortical thickness*

Different degrees of cortical thinning were predominantly identified in fronto-temporalareas, including the temporal pole (right: 0.010; left: 0.023), the planum polare (right:0.010; left: 0.009) and the planum temporale (right: 0.014; left: 0.015), the entorhinalarea (right: 0.019; left: 0.022), the middle cingulate gyrus (right: 0.015; left: 0.015), thesuperior temporal gyrus (right: 0.017; left: 0.018), the left middle frontal gyrus (1.197),the triangular inferior frontal gyrus (right: 0.013; left: 0.013), the lateral orbital gyrus(right: 0.018; left: 0.011), the supplementary motor cortex (right: 0.015; left: 0.018), thefrontal operculum (right: 0.018; left: 0.016), the precuneus (right: 0.017; left: 0.017) andthe supramarginal gyrus (right: 0.012; left: 0.016).

##### Leukoencephalopathy and subependymal nodules. 

The severity of leukoencephalopathy was evaluated using the Fazekas scale [44]. The score was 4 (3, irregular periventricular signal extending into the deep white matter + 1, punctate foci for deep white matter), with no involvement of subcortical U-fibers or the striatum. Ten subependymal nodules were identified, most of which small, with a nodular appearance and located in correspondence of the lateral ventricles, predominantly at the level of the mid-body bilaterally, of the frontal horn of the left ventricle, and the trigone of the right ventricle.

##### MRI longitudinal comparison.

**Fig. 3:**
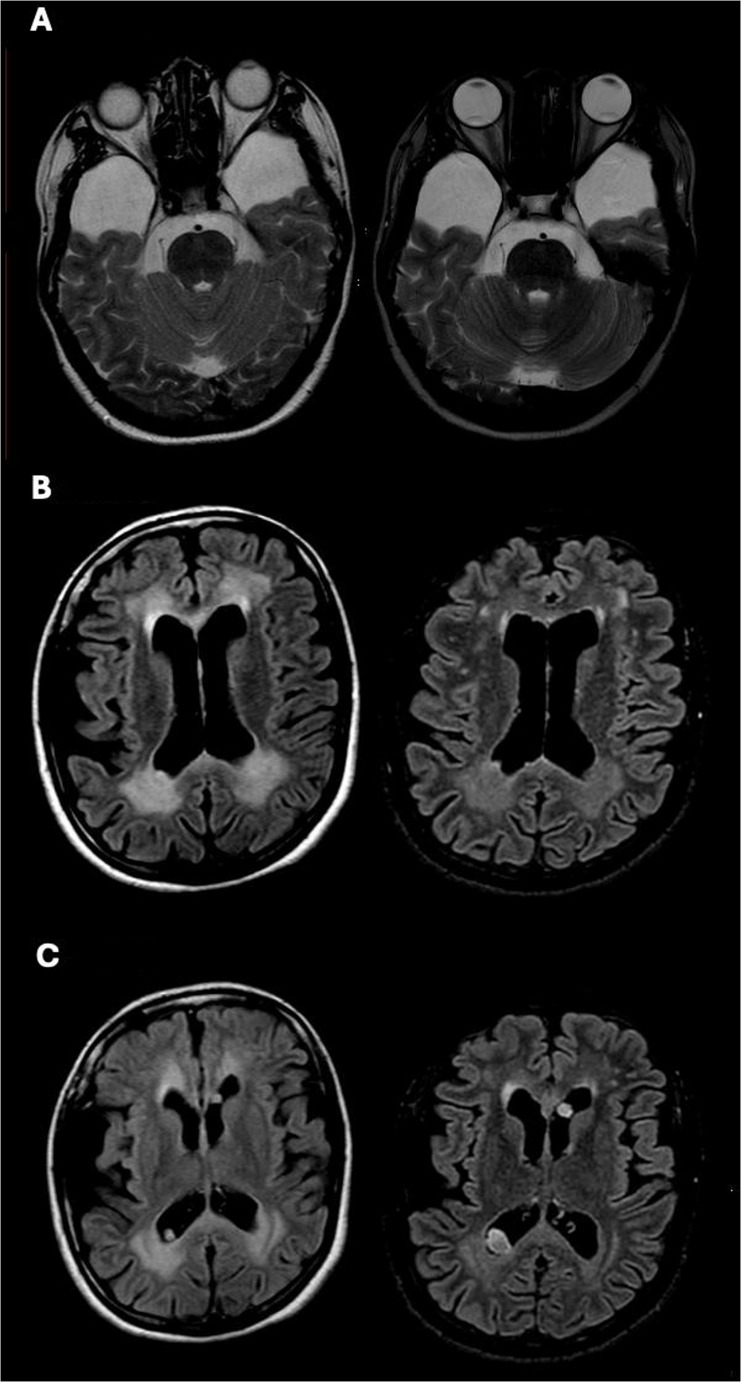
Panel (**A**) T2-weighted axial sections showing bilateral temporal hypoplasia. Panel (**B**) T2-weighted FLAIR axial sections demonstrating a reduction of altered signal in the frontal horns in the late control (right). Panel (**C**) T2-weighted FLAIR axial images showing two of the ten subependymal nodules, one located the right trigone (radiological convention) and one in the mid-body of the left lateral ventricle, increasing in size at the second control (right)

The 1T MRI scan executed 10 years earlier was not suitable for performing volumetric analyses. A qualitative neuroradiological comparison indicated that there were no obvious differences between the two scans in terms of hypoplasia/hypotrophy (see Fig. 3, panel A).

Interestingly, a reduction was observed in the extent of anterior (frontal) periventricular white matter hyperintensities in the second MRI, which did not result however in a decrease in the Fazekas score, at variance with the paratrigonal region, which remained unchanged (Fig. 3, panel B). In both scans, diffusion-weighted imaging (DWI) sequences consistently showed facilitation, namely increased water molecule movement, in correspondence with the white matter lesions.

Subependymal nodules remained stable in number; however, two of the larger nodules had increased in size at the second imaging study (Fig. 3, panel C). One nodule, located in the mid-body of the left lateral ventricle, with the longest axis increasing from 10 to 15 mm, also changed in appearance from a purely nodular to a partially cystic morphology. Another nodule, located in the trigone of the right lateral ventricle, increased from 6 mm to 13 mm.

#### Psychometric assessment

All scores in neuropsychological tests are shown in Table 3. During cognitive testing the patient showed adequate levels of sustained attention and collaboration. She performed within normal limits in language tasks. However, defective performances were observed in verbal short-term memory (forward digit span) [47] and in the verbal learning tests (immediate prose recall) [48], but not in verbal working memory (backward digit span) [47]. The poor performance in verbal short-term memory was confirmed at the MoCA subtests, in which the patient was unable to repeat correctly four numbers in the same order and the second, longer sentence, while she correctly repeated the first one. Non-verbal short-term and working memory performances fell within normal ranges.

In the immediate and delayed recall of the Rey Auditory Verbal Learning Test [48], the serial position effects, which refer to the order in which words from list learning tasks are recalled, were evaluated using the corrected regional scoring, which accounts for the number of items in each region (primacy, middle, recency) and the total number of words recalled [49]. The patient showed a strong primacy and a less prominent recency effects: First repetition: primacy 0.66, middle 0.00, recency 0.20; 1–5 immediate recall: primacy 0.93, middle 0.34, recency 0.60; delayed recall: primacy 1.00, middle 0.57, recency 0.60. This result is in line with the present evidence of a verbal short-term memory deficit. Patients with a selective deficit of auditory verbal span, also show a reduced recency effect [50].

Defective performances were also found in tests assessing verbal inferential-executive abilities based on previously acquired semantic knowledge as the Cognitive Estimation Task [51] for both absolute and bizarreness errors, while semantic association (Semantic Association Test) [52] was preserved. Performances in all the other cognitive domains assessed (praxis, spatial cognition, logical-deductive abstract reasoning, non-verbal executive functioning, verbal and non-verbal long-term memory) fell within normal ranges, adjusted for age and education.

**Table 3:** Neuropsychological data. Raw, adjusted, and equivalent scores or cut-off scores are reported

Neuropsychological evaluation				
**Montreal Cognitive Assessment-MoCA**	RS	Adj	AS	ES
*Visuospatial /Executive*	5/5			
*Naming*	3/3			
*Attention:*				
Digit Span	1/2			
Sustained attention	1/1			
Series of 7	3/3			
*Language:*				
Repetition	1/2			
Fluency	1/1			
Abstract ion	2/2			
*Delayed recall*	4/5			
*Orientation*	6/6			
Tot. MoCA:	**27/30**	-1.22	25.78	4
VS	4/4		4.00	4
EF	4/4		4.00	4
L	4/5	-0.15	3.85	2
A	5/6	-0.12	4.88	2
M	4/5	-0.80	3.20	4
O	6/6		6.00	4
AES				3.33
**Clock drawing te** **st**	10/10			8
**Language**	**RS**	**Adj**	**AS**	**ES**
* Token test*	34/36	-1.25	32.75	3
* Visual confrontation naming*	47/48	-0.06	46.94	3
* Semantic fluency*	37	-4.22	32.78	2
* Semantic Association Test*				
* SAT overall*	75/76	-0.06	74.94	4
* CA*	19/19		19	4
* EA*	18/19	0.12	18.12	3
FA	19/19		19	4
* VEA*	19/19		19	4
**Praxies**	**RS**	**Adj**	**AS**	**ES/Cut-off**
* Rey–Osterrieth complex figure copy*	30/36	-1.10	28.90	1
* Ideomotor apraxia – right arm*	71/72			52
* Ideomotor apraxia – left arm*	72/72			52
**Spatial Cognition**	**RS**			**Cut-off**
*Apple cancellation*				
Accuracy score - Total omission errors	2/50			5
Asymmetry score for egocentric neglect—omission error difference	-1			2
Asymmetry score for allocentric neglect—omission error difference	0			2
Execution time	104			170
* Line Bisection*	-2.87 %			-8.17 < x < 5.75
**Abstract reasoning**	**RS**	**Adj**	**AS**	**ES**
* Raven’s Standard Progressive Matrices*	38/48	-7.50	30.50	4
**Executive Functions**	**RS**	**Adj**	**AS**	**ES/Cut-off**
* Frontal Assessment Battery*				
Total raw score	16/18	-0.46	15.54	3
FAB1	4/6	-0.05	3.95	1
FAB2	6/6		6.00	4
FAB3	6/6		6.00	4
*Wisconsin Card Sorting Test*				
Perseverations	9	-1.34	7.66	4
Non-perseverative errors	7	0.12	7.12	4
Failures	0			4
Global value	22	-0.12	21.88	4
*Trail Making Test*				
Section A	29	1	30	4
Section B	59	-5	54	4
Difference (B-A)	30	-6	24	4
* Phonemic fluency*	21	2.73	23.73	2
*Stroop test*				
Time	17.5	6.95	24.45	3
Errors	1.5	0.60	2.10	2
*Cognitive Estimation Task (CET)*				
Absolute errors	20/42	-0.80	19.20	< 18
Bizarreness	11/21			< 4
**Short-term memory**	**RS**	**Adj**	**AS**	**ES**
* Forward Digit span*	4	-0.25	3.75	**0**
* Forward Corsi span*	5	-0.21	4.79	2
**Working memory**	**RS**	**Adj**	**AS**	**ES**
* Backward Digit span*	5	-0.17	4.83	4
* Backward Corsi span*	6	-0.63	5.37	4
**Long-term memory**	**RS**	**Adj**	**AS**	**ES/Cut-off**
*Rey-Auditory Verbal Learning test*				
Immediate recall	41/75	-5.02	35.98	2
Delayed recall	10/15	-1.78	8.22	3
Correct recognitions	13/15			
False recognitions	1/30			
*Prose memory*				
Immediate recall- Hierarchical score	17.5/75.5	-9.50	8.00	**0**
Delayed recall- Hierarchical score	13/75.5			
Index of mnesic decline	25.71%			53%
* Rey–Osterrieth complex figure delayed recall*	24/36	-2.00	22.00	4
**Mood**	**RS**			**Cut-off**
* Beck Depression Inventory-II*	9/63			5-9

*RS *raw score; *Adj *adjustment coefficient for age, education, sex; *AS *adjusted score; *ES *equivalent score, expressing raw scores, adjusted for gender, age and education, ranging from ‘0’ (defective performance), ‘1’ (borderline performance), and ‘2’ to ‘4’ (performance within normal range) [53]. Defective performance: Bold score

## Discussion

GA1 patients with onset of the disease after 6 years of age are defined as LO. They represent a minority of the GA1 patients [12] and whether they can be considered as a distinct variant of the disease is a matter of discussion [15]. Our scoping review points out that LO patients have had symptoms even years before their diagnosis, proving the difficulties met by clinicians, being this a rare, multifaceted and phenotypically variable disease. Interestingly, although they usually have insidious onset of symptoms, no acute encephalopathic crises have been reported in the literature so far [15]. Here, we described the clinical features of a novel, putatively asymptomatic GA1 adult case identified through the NS of her healthy baby, who, 10 years before coming to our attention, had had an acute severe neurological crisis (hyposthenia and walking impairment) occurring during an infection, with symptoms disappearing after about 14 days.

The most frequent finding in GA1 LO patients are macrocephaly and MRIs typical abnormalities (Tables 1 and 2) - frontotemporal hypoplasia, abnormal signal of the white matter and subependymal nodules- which might be a strong hint to diagnosis.

Notably, the present scoping review showed that no volumetric and cortical thickness measurements have been performed in LO patients so far. In the patient reported here, neuroradiological MRI analysis confirmed the presence of fronto-temporal hypoplasia, leukoencephalopathy and subependymal nodules, as typically reported in LO GA1 patients [2, 12]. The morphometric study, performed for the first time in a LO GA1 patient, highlighted, compared to matched controls, abnormally reduced volumes and cortical thinning, mainly involving fronto-temporal areas, as the temporal pole, the planum polare, the medial frontal cortex bilaterally, and the left lateral orbital gyrus. Parietal and occipital regions were less involved (see Fig. 2 and Tables S1 and S2, for volumes and cortical thickness results, respectively). At a qualitative inspection, after 10 years, cortical hypotrophy appeared to have remained grossly unchanged overtime, although it cannot be ruled out that a proper quantitative comparison might have revealed some slight changes in cortical thickness.

A recent study found different degrees of cortical thinning in grossly overlapping areas, compared to the ones described here, also in a group of 17 early-onset patients (mean age 38 ± 17 months) [30]. Brain hypotrophy, as well as fronto-temporal hypoplasia, at least in the early adult phase of life, might represent a consequence of a pathological organization of brain development, characterized in children by deviations from the normal maturational changes of the developing brain, such as immature gyral pattern and myelination delay, which, in some cases, may regress with maturation (see case 3 in Harting et al. [2], who showed a regression of extra-striatal abnormalities within the first four years).

The more provisional picture due to brain development in children is further characterized by areas of increased cortical thickness, as found by Bian and colleagues [30]. In their group of early-onset patients, increased cortical thickness (in the precentral and postcentral, parahippocampal, lingual, superior frontal, middle and inferior temporal gyri, and in the precuneus) was interpreted as a cortical reorganization supporting functional recovery during abnormal development. In the present study we did not find higher cortical thickness in specific brain areas (see Table S2). In our adult patient, it is then possible that brain reorganization had concluded, manifesting predominantly as signs of cortical thinning. Further longitudinal studies are needed to confirm the present observations and to investigate the relationship between aberrant development and neurodegeneration [54].

As for the other neuroradiological signs typical of LO GA1, we found 10 subependymal nodules localized in periventricular areas, at the level of the ventricular roof and the septum pellucidum, which remained stable in number but increased in volume overtime (Fig. 3, panel C), with one evolving from a nodular to a cystic aspect, as typically reported [2, 15].

Interestingly, there was a reduction of white matter hyperintensities after 10 years, particularly in the periventricular areas surrounding the frontal horns of the lateral ventricles, compared to the paratrigonal region, that, conversely, remained grossly unchanged. This result was somewhat unexpected since white matter abnormalities in LO GA1 are considered to remain stable or slightly worsen overtime [2, 15, 55–57] (see also Boy et al. [12]). In both the available imaging, DWI sequences showed facilitation at the site of white matter lesions. Restricted diffusion is a sign of an ongoing acute crisis [58]. However, the first brain MRI was performed about three weeks after that, thus in a post-acute/chronic phase, when facilitated diffusion is expected [59]. In sum, the putative mechanism underlying the WM hyperintensity reduction remain uncertain.

Cognitive symptoms are sometimes reported in GA1 patients, such as mild cognitive delay in children and dementia in adults [2, 6, 7, 9, 10, 12, 20, 28, 34]. Other patients have been reported with intelligence/cognition within the normal range [4, 7, 11–15, 17, 19, 33], although neuropsychological assessments were often limited to clinical judgement and descriptive commentaries or to the administration of screening tests (see Tables 1 and 2). In patients diagnosed by symptoms after 6 years of age, exceptions are the study by Fernandez-Alvarez et al. [6] in a 16-years-old patient, who presented with slight difficulties in attention, execution, and fine motor function, and the one by Gelener and colleagues [13], who assessed various cognitive domains, as processing speed, long-term memory, word retrieval, visuospatial processing, and executive functions in a 35-year-old Turkish Cypriot GA1 female. In this case, all the patient’s scores were found to be within the normal range, although mild stress-related anxiety and depression were detected through standardized scales. To date, the case described here is the first undiagnosed LO cases detected through neonatal or family screening with an extensive neuropsychological evaluation.

As shown in Table 3, in our patient, the neuropsychological assessment revealed mild difficulties in verbal executive and memory tasks. Specifically, defective scores were observed in verbal short-term memory (span: forward repetition of digit sequences limited to 4) and immediate prose recall. These findings suggest a deficit of the phonological short-term memory system [60]. Conversely, the unimpaired performances in forward and backward spatial span indicates a normal operation of the nonverbal component of short-term memory, the visuo-spatial sketch pad (see, e.g., Baddeley [61]). Backward repetition span is not usually investigated in patients with a deficit of phonological short-term memory [62]. Forward digit span is higher than backward digit span in most (92.9% in a sample of 1250 participants by Wilde et al. [63]), but not all, neurologically unimpaired participants. Both spans, but more backward than forward span, are considered to rely on central executive/working memory resources. These systems may however not overlap and be distinct [64] accounting for the dissociation found in this study. Concerning the neural substrates, there is evidence that the left posterior-superior temporal cortex, and specifically the supramarginal gyrus of the left inferior parietal lobule, play a relevant role, being involved in the execution of verbal span tasks, functionally based on the operation of verbal, phonological short-term memory [65, 66]. Interestingly, a reduction in the volume of the supramarginal gyrus was found in the patient described here.

The present LO GA1 patient exhibited also poor verbal executive functions, in terms of verbal cognitive estimation abilities. Cognitive and magnitude estimations are known to be grounded on frontal-parietal networks [67], with the right middle and inferior frontal gyrus being related with the tendency to give extreme responses [68]. Also, the right anterior temporal lobe contributes to magnitude knowledge [69]. Interestingly, no signs of semantic deficit (non-verbal semantic association) were found. In line with these considerations, the patient presented aberrant development of the temporal pole and fronto-temporal atrophy.

Previous studies investigating cognitive performances in LO patients reported, in some cases, the involvement of executive functions, although often not in detail. A 56 years-old male showed impaired executive and working memory functions and difficulties in learning new information (MMSE score = 25/30). The MRI showed signs of communicating hydrocephalus, bilateral frontotemporal atrophy and prominent periventricular and deep leukodystrophy [9]. A 32 years-old female exhibited reduced verbal fluency and motor sequencing, associated with confluent symmetric bilateral WM signal abnormalities and mild atrophy at the MRI [39]. In a 16 years-old girl, a neuropsychological assessment showed slight difficulties in attention, execution and fine motor function, associated with MRI-assessed damage to the putamen and diffuse bilateral frontal WM abnormalities [6]. Importantly, no previous studies described in some detail a deficit of phonological short-term memory (digit span) in a LO GA1 patient.

A relevant point concerns the discrepancy between the extent of the brain involvement and the slight cognitive deficits described (when found) in GA1 patients, both in infants and in adults, even when assessed through an in-depth neuropsychological evaluation. It is well known that the developing brain has a remarkable capacity for reorganization due to plasticity, which may sustain the development of cognitive functions [70, 71]. Plasticity, if indeed present, does not appear to be able to prevent cortical thinning. The developmental features of the disorder may tentatively account for the behavioral-anatomical discrepancy mentioned above, in terms of a functional adaptation to slowly developing brain abnormalities. The same picture found in adults seems to further support the neurodevelopmental nature of the disorder [72].

A limitation of this study relates to the use of an automated morphometric assessment. Automated pipelines such as Vol2Brain are primarily trained and validated on anatomically normal brains; therefore, in the presence of congenital brain malformations, including temporal lobe hypoplasia, segmentation accuracy at the regional level may be reduced and volumetric results should be interpreted with caution, particularly in single-case studies. Notwithstanding these limitations, Vol2Brain has demonstrated acceptable reliability for gray matter volumes, comparable to other widely used segmentation tools [73], thus, when interpreted in the context of this methodological limitation, can be considered a suitable tool for volumetric assessment. Furthermore, the use of different types of scans did not permit a quantitative comparison between the two MRI imaging over 10 years: although at a qualitative inspection, cortical hypotrophy appeared to have remained unchanged, it cannot be ruled out that a proper quantitative comparison might have revealed some changes in cortical thickness.

## Conclusion

 To conclude, in the present case brain morphometric analysis revealed diffuse hypotrophy predominantly in, but not limited to, fronto-temporal regions. An extensive neuropsychological assessment showed mild difficulties in verbal executive functions (inferential processes) and phonological short-term memory. Our case description and scoping review may serve as a hint to proceed with more systematic and longitudinal studies in LO GA1, in which both neuroanatomical and neuropsychological deep investigations should be integrated, contributing to a longitudinal monitoring of such patients and to unveiling the complex ethio-pathogenesis of the disease. The number of females identified through the neonatal screening of their healthy infants is increasing and this is really an extra-benefit obtained through the expanded newborn screening. Among the many unanswered questions about GA1, there is the effect of regular L-carnitine supplementation in preventing clinical and neuroradiological worsening overtime. Indeed, while low lysine diet and L-carnitine supplementation have been shown to be effective in improving the neurological outcome of affected newborns [74] there is not enough evidence to confirm their efficacy in LO patients. Due to the rarity of the LO form of GA1, multicenter studies might be useful to reach GA1 open questions.

## Electronic Supplementary Material

Below is the link to the electronic supplementary material.

Supplementary Material 1
