# Supplementary material for: Brain morphometry and cognition in late-onset glutaric aciduria type 1: scoping review and novel insights from a case report
Source: Neurol Sci. 2026 Mar 4;47(4):319. doi: 10.1007/s10072-026-08886-9 (PMC12960389; doi:10.1007/s10072-026-08886-9)
Supplement: Supplementary file 1 — Supplementary Material 1 [file 10072_2026_8886_MOESM1_ESM.pdf]

Neuropsychological Assessment

The following cognitive domains have been investigated:

i) Language: verbal comprehension (Token Test [1], visual confrontation naming [2], semantic fluencies [3], and semantic memory (Semantic Association Test; SAT [4]);

ii) Praxis: constructive (Rey–Osterrieth complex figure copy [5] and left-and right-arm ideomotor praxis [6];

iii) Spatial cognition: visuo-spatial exploration (apple cancellation [7] and length estimation (line bisection; [8]);

iv) Logical-deductive abstract reasoning (Raven's Standard Progressive Matrices [1]);

v) Executive functioning: Frontal Assessment Battery (FAB [9]), problem solving (Wisconsin Card Sorting Test [10]), selective attention and set-shifting (Trial Making Test [11]), verbal flexibility (phonemic fluencies [3]), interference control (Stroop test [12]), and inferential thinking (Cognitive Estimation Test, CET [13]);

vi) Short-term and working memory, verbal and visuo-spatial: forward and backward Digit and Corsi Span [14];

vii) Long-term memory: words (Rey-Auditory Verbal Learning test, RAVLT [15]), prose [16], non-verbal material (delayed recall of the Rey-Osterrieth Complex Figure [5]).

Table S1. Volumes of the whole brain subregions and expected 95% limits of normalized volume in function of sex and age for each measure. Light grey: measures lower than expected; dark grey: measures higher than expected.

VOLUME	TOTAL				RIGHT				LEFT			
**Structures**	**Patient cm3**	**Patient vol%**	**Inferior Normative Value**	**Superior Normative Value**	**Patient cm3**	**Patient vol%**	**Inferior Normative Value**	**Superior Normative Value**	**Patient cm3**	**Patient vol%**	**Inferior Normative Value**	**Superior Normative Value**
**Accumbens**	0,566	0,035	0,034	0,063	0,248	0,015	0,015	0,031	0,318	0,020	0,018	0,034
**Amygdala**	1,431	0,089	0,116	0,171	0,682	0,042	0,059	0,086	0,749	0,046	0,056	0,087
**Basal Forebrain**	1,037	0,064	0,037	0,067	0,546	0,034	0,018	0,035	0,492	0,030	0,017	0,034
**Caudate**	7,349	0,456	0,443	0,663	3,690	0,229	0,224	0,334	3,660	0,227	0,215	0,333
**Hippocampus**	8,078	0,501	0,503	0,683	4,207	0,261	0,253	0,345	3,871	0,240	0,246	0,342
**Pallidum**	3,195	0,198	0,163	0,256	1,621	0,101	0,080	0,129	1,574	0,098	0,081	0,129
**Putamen**	7,814	0,485	0,522	0,752	3,915	0,243	0,257	0,375	3,899	0,242	0,263	0,379
**Thalamus**	12,758	0,791	0,780	1,041	6,454	0,400	0,388	0,514	6,304	0,391	0,390	0,530
**Ventral DC**	11,941	0,741	0,631	0,814	5,839	0,362	0,313	0,405	6,102	0,379	0,317	0,410
**Frontal lobe**	169,026	10,486	12,133	14,779	83,852	5,202	6,027	7,370	85,174	5,284	6,078	7,438
**Frontal pole**	7,410	0,460	0,375	0,612	3,489	0,216	0,179	0,308	3,921	0,243	0,183	0,311
**Gyrus rectus**	4,405	0,273	0,223	0,364	2,286	0,142	0,112	0,195	2,119	0,131	0,102	0,178
**Opercular inf. frontal gyrus**	7,199	0,447	0,404	0,639	3,163	0,196	0,191	0,336	4,036	0,250	0,184	0,332
**Orbital inf. frontal gyrus**	2,424	0,150	0,122	0,299	1,108	0,069	0,057	0,162	1,316	0,082	0,044	0,156
**Triangular inf. frontal gyrus**	5,463	0,339	0,406	0,669	2,985	0,185	0,190	0,352	2,478	0,154	0,190	0,344
**Medial frontal cortex**	1,500	0,093	0,184	0,310	0,708	0,044	0,086	0,173	0,791	0,049	0,078	0,156
**Middle frontal gyrus**	40,619	2,520	2,593	3,391	21,328	1,323	1,270	1,739	19,291	1,197	1,286	1,689
**Anterior orbital gyrus**	3,890	0,241	0,203	0,343	1,874	0,116	0,101	0,185	2,016	0,125	0,087	0,173
**Lateral orbital gyrus**	2,451	0,152	0,221	0,413	1,700	0,105	0,104	0,219	0,751	0,047	0,099	0,211
**Medial orbital gyrus**	10,813	0,671	0,500	0,721	5,194	0,322	0,234	0,359	5,619	0,349	0,250	0,375

**Posterior orbital gyrus**	5,042	0,313	0,325	0,541	2,419	0,150	0,152	0,272	2,623	0,163	0,159	0,283
**Precentral gyrus**	22,999	1,427	1,667	2,200	10,943	0,679	0,819	1,108	12,056	0,748	0,822	1,118
**Precentral gyrus medial segment**	3,538	0,219	0,323	0,486	1,529	0,095	0,149	0,256	2,009	0,125	0,155	0,249
**Subcallosal area**	3,459	0,215	0,116	0,248	1,857	0,115	0,055	0,126	1,602	0,099	0,059	0,125
**Sup. frontal gyrus**	28,407	1,762	1,858	2,553	14,303	0,887	0,898	1,288	14,104	0,875	0,920	1,305
**Sup. frontal gyrus medial segment**	11,620	0,721	0,835	1,214	4,911	0,305	0,403	0,662	6,710	0,416	0,383	0,601
**Supplementary motor cortex**	7,788	0,483	0,631	0,940	4,056	0,252	0,301	0,475	3,732	0,232	0,304	0,491
**Temporal lobe**	93,741	5,815	6,743	8,333	47,587	2,952	3,340	4,147	46,154	2,863	3,359	4,230
**Fusiform gyrus**	19,573	1,214	0,952	1,397	10,315	0,640	0,457	0,709	9,257	0,574	0,468	0,714
**Planum polare**	2,662	0,165	0,248	0,356	1,037	0,064	0,117	0,176	1,625	0,101	0,122	0,188
**Planum temporale**	2,998	0,186	0,210	0,377	1,491	0,093	0,091	0,183	1,507	0,093	0,102	0,211
**Inf. temporal gyrus**	27,977	1,736	1,400	2,006	13,476	0,836	0,669	1,019	14,500	0,900	0,690	1,027
**Middle temporal gyrus**	28,296	1,755	1,818	2,478	14,338	0,889	0,914	1,275	13,959	0,866	0,864	1,242
**Sup. temporal gyrus**	9,123	0,566	0,889	1,253	5,188	0,322	0,445	0,660	3,935	0,244	0,410	0,628
**Transverse temporal gyrus**	3,053	0,189	0,154	0,309	1,740	0,108	0,065	0,151	1,313	0,081	0,078	0,170
**Temporal pole**	0,059	0,004	1,051	1,595	0,001	0,000	0,544	0,819	0,058	0,004	0,490	0,793
**Parietal lobe**	92,082	5,712	7,422	9,089	44,453	2,758	3,677	4,533	47,630	2,955	3,708	4,592
**Angular gyrus**	21,902	1,359	1,157	1,824	10,119	0,628	0,614	0,975	11,783	0,731	0,510	0,883
**Postcentral gyrus**	18,932	1,175	1,229	1,699	9,365	0,581	0,582	0,854	9,567	0,594	0,615	0,877
**Postcentral gyrus medial segment**	1,114	0,069	0,088	0,170	0,673	0,042	0,037	0,092	0,440	0,027	0,040	0,089
**Precuneus**	17,099	1,061	1,413	1,932	9,030	0,560	0,694	0,984	8,069	0,501	0,695	0,972
**Sup. parietal lobule**	18,250	1,132	1,258	1,763	8,725	0,541	0,597	0,886	9,525	0,591	0,628	0,909
**Supramarginal gyrus**	14,786	0,917	1,049	1,493	6,541	0,406	0,503	0,772	8,245	0,511	0,503	0,764

**Occipital lobe**	69,052	4,284	4,548	6,118	36,518	2,266	2,292	3,111	32,534	2,018	2,192	3,072
**Calcarine cortex**	4,811	0,298	0,299	0,623	2,538	0,157	0,142	0,317	2,273	0,141	0,145	0,318
**Cuneus**	6,982	0,433	0,497	0,770	3,584	0,222	0,235	0,394	3,398	0,211	0,236	0,403
**Lingual gyrus**	16,249	1,008	0,986	1,450	8,037	0,499	0,473	0,740	8,211	0,509	0,491	0,732
**Occipital fusiform gyrus**	7,038	0,437	0,336	0,621	4,096	0,254	0,157	0,323	2,942	0,183	0,157	0,319
**Inf. occipital gyrus**	12,534	0,778	0,708	1,085	7,265	0,451	0,327	0,560	5,269	0,327	0,345	0,561
**Middle occipital gyrus**	12,154	0,754	0,570	0,924	6,265	0,389	0,259	0,444	5,889	0,365	0,282	0,510
**Sup. occipital gyrus**	5,758	0,357	0,425	0,671	3,031	0,188	0,217	0,358	2,727	0,169	0,187	0,341
**Occipital pole**	3,527	0,219	0,223	0,462	1,703	0,106	0,094	0,216	1,824	0,113	0,109	0,267
**Limbic cortex**	31,859	1,976	2,687	3,430	14,962	0,928	1,335	1,762	16,897	1,048	1,301	1,720
**Entorhinal area**	2,236	0,139	0,217	0,340	0,927	0,057	0,107	0,173	1,309	0,081	0,102	0,175
**Anterior cingulate gyrus**	10,100	0,627	0,694	1,030	5,155	0,320	0,309	0,533	4,944	0,307	0,340	0,543
**Middle cingulate gyrus**	7,539	0,468	0,659	0,938	3,348	0,208	0,325	0,481	4,191	0,260	0,308	0,483
**Posterior cingulate gyrus**	7,462	0,463	0,577	0,805	3,646	0,226	0,271	0,406	3,816	0,237	0,289	0,416
**Parahippocampal gyrus**	4,522	0,281	0,345	0,512	1,886	0,117	0,160	0,251	2,636	0,164	0,176	0,266
**nsular cortex**	28,087	1,742	1,951	2,560	13,525	0,839	0,989	1,314	14,563	0,903	0,948	1,259
**Anterior insula**	7,983	0,495	0,521	0,754	4,185	0,260	0,255	0,379	3,798	0,236	0,260	0,381
**Posterior insula**	5,855	0,363	0,273	0,429	3,036	0,188	0,136	0,221	2,819	0,175	0,131	0,214
**Central operculum**	7,406	0,459	0,517	0,717	3,298	0,205	0,247	0,367	4,108	0,255	0,254	0,366
**Frontal operculum**	2,924	0,181	0,243	0,384	1,402	0,087	0,110	0,191	1,522	0,094	0,116	0,209
**Parietal operculum**	3,920	0,243	0,238	0,436	1,605	0,100	0,094	0,208	2,316	0,144	0,129	0,243
**Inf. Lateral Ventricle**	1,540	0,095	0,000	0,070	1,180	0,073	0,000	0,042	0,360	0,022	0,000	0,033
**Lateral Ventricle**	65,560	4,067	0,000	2,075	32,590	2,022	0,000	1,028	32,970	2,064	0,000	1,102

Table S2. Cortical thickness values of the whole brain subregions and expected 95% limits of normalized thickness in function of sex and age for each measure. Light grey: measures lower than expected; dark grey: measures higher than expected.

CORTICAL THICKNESS	TOTAL				RIGHT				LEFT			
Structures	Patient mm	Patient vol%	Inferior Normative Value	Superior Normative Value	Patient mm	Patient vol%	Inferior Normative Value	Superior Normative Value	Patient mm	Patient vol%	Inferior Normative Value	Superior Normative Value
Frontal lobe	2,122	0,018	0,019	0,027	2,127	0,018	0,019	0,027	2,116	0,018	0,019	0,027
Frontal pole	2,233	0,019	0,018	0,030	2,298	0,020	0,018	0,031	2,175	0,019	0,017	0,029
Gyrus rectus	3,438	0,029	0,019	0,039	3,793	0,032	0,019	0,040	3,054	0,026	0,018	0,040
Opercular inf. frontal gyrus	1,749	0,015	0,017	0,025	1,807	0,015	0,017	0,025	1,703	0,015	0,016	0,026
Orbital inf. frontal gyrus	1,688	0,014	0,018	0,028	1,992	0,017	0,018	0,029	1,432	0,012	0,017	0,028
Triangular inf. frontal gyrus	1,528	0,013	0,018	0,026	1,573	0,013	0,018	0,027	1,473	0,013	0,017	0,026
Medial frontal cortex	2,966	0,025	0,022	0,034	3,059	0,026	0,022	0,034	2,884	0,025	0,021	0,034
Middle frontal gyrus	2,123	0,018	0,019	0,030	2,168	0,018	0,019	0,030	2,074	0,018	0,019	0,030
Anterior orbital gyrus	2,286	0,019	0,022	0,034	2,464	0,021	0,021	0,034	2,120	0,018	0,022	0,035
Lateral orbital gyrus	1,838	0,016	0,020	0,032	2,068	0,018	0,021	0,032	1,318	0,011	0,019	0,033
Medial orbital gyrus	3,231	0,028	0,019	0,033	3,410	0,029	0,018	0,034	3,066	0,026	0,019	0,033
Posterior orbital gyrus	2,631	0,022	0,023	0,036	2,698	0,023	0,023	0,037	2,570	0,022	0,022	0,036
Precentral gyrus	1,455	0,012	0,014	0,020	1,328	0,011	0,014	0,020	1,570	0,013	0,014	0,020
Precentral gyrus medial segment	1,141	0,010	0,013	0,022	1,120	0,010	0,013	0,022	1,157	0,010	0,013	0,023
Subcallosal area	3,066	0,026	0,010	0,033	2,875	0,025	0,008	0,033	3,288	0,028	0,011	0,033
Sup. frontal gyrus	1,981	0,017	0,017	0,025	1,936	0,017	0,017	0,026	2,027	0,017	0,017	0,025
Sup. frontal gyrus medial segment	2,604	0,022	0,023	0,032	2,411	0,021	0,023	0,033	2,746	0,023	0,022	0,032
Supplementary motor cortex	1,925	0,016	0,019	0,029	1,773	0,015	0,019	0,029	2,091	0,018	0,019	0,029
Temporal lobe	3,237	0,028	0,015	0,023	3,085	0,026	0,015	0,023	3,394	0,029	0,015	0,023

**Fusiform gyrus**	4,251	0,036	0,027	0,039	4,276	0,036	0,026	0,039	4,224	0,036	0,027	0,040
**Planum polare**	1,096	0,009	0,013	0,022	1,153	0,010	0,012	0,022	1,060	0,009	0,012	0,023
**Planum temporale**	1,670	0,014	0,016	0,025	1,587	0,014	0,015	0,025	1,753	0,015	0,016	0,026
**Inf. temporal gyrus**	3,745	0,032	0,025	0,037	3,565	0,030	0,024	0,037	3,912	0,033	0,025	0,038
**Middle temporal gyrus**	2,985	0,025	0,024	0,033	2,679	0,023	0,024	0,033	3,299	0,028	0,024	0,034
**Sup. temporal gyrus**	2,053	0,018	0,020	0,027	2,012	0,017	0,019	0,027	2,108	0,018	0,020	0,028
**Transverse temporal gyrus**	1,372	0,012	0,015	0,025	1,292	0,011	0,014	0,025	1,479	0,013	0,015	0,026
**Temporal Pole**	2,715	0,023	0,027	0,039	1,172	0,010	0,027	0,039	2,740	0,023	0,026	0,040
**Parietal lobe**	1,413	0,012	0,025	0,033	1,312	0,011	0,025	0,034	1,506	0,013	0,024	0,033
**Angular gyrus**	1,619	0,014	0,018	0,027	1,465	0,012	0,017	0,028	1,752	0,015	0,017	0,028
**Postcentral gyrus**	0,881	0,008	0,009	0,016	0,812	0,007	0,009	0,016	0,948	0,008	0,009	0,016
**Postcentral gyrus medial segment**	0,644	0,005	0,006	0,016	0,670	0,006	0,005	0,016	0,604	0,005	0,005	0,016
**Precuneus**	1,997	0,017	0,018	0,030	1,993	0,017	0,018	0,030	2,000	0,017	0,018	0,030
**Sup. parietal lobule**	0,999	0,009	0,010	0,019	0,940	0,008	0,009	0,018	1,053	0,009	0,010	0,020
**Supramarginal gyrus**	1,680	0,014	0,017	0,026	1,416	0,012	0,016	0,026	1,890	0,016	0,016	0,027
**Occipital lobe**	2,140	0,018	0,015	0,025	2,088	0,018	0,015	0,025	2,200	0,019	0,014	0,025
**Calcarine cortex**	1,295	0,011	0,008	0,023	1,213	0,010	0,008	0,023	1,386	0,012	0,008	0,023
**Cuneus**	1,258	0,011	0,010	0,022	1,286	0,011	0,010	0,022	1,228	0,010	0,010	0,022
**Lingual gyrus**	2,626	0,022	0,016	0,030	2,450	0,021	0,016	0,030	2,799	0,024	0,017	0,030
**Occipital fusiform gyrus**	2,660	0,023	0,015	0,028	2,539	0,022	0,015	0,029	2,830	0,024	0,014	0,028
**Inf. occipital gyrus**	2,568	0,022	0,017	0,027	2,547	0,022	0,016	0,027	2,598	0,022	0,018	0,028
**Middle occipital gyrus**	2,139	0,018	0,018	0,030	2,105	0,018	0,018	0,030	2,175	0,019	0,018	0,030
**Sup. occipital gyrus**	1,229	0,010	0,011	0,020	1,302	0,011	0,011	0,020	1,148	0,010	0,010	0,020
**Occipital pole**	1,739	0,015	0,007	0,018	1,661	0,014	0,007	0,018	1,812	0,015	0,006	0,019
**Limbic cortex**	2,429	0,021	0,024	0,034	2,437	0,021	0,024	0,034	2,423	0,021	0,025	0,034
**Entorhinal area**	2,483	0,021	0,025	0,034	2,280	0,019	0,024	0,034	2,626	0,022	0,023	0,034

**Anterior cingulate gyrus**	2,979	0,025	0,027	0,039	3,171	0,027	0,027	0,040	2,779	0,024	0,027	0,040
**Middle cingulate gyrus**	1,758	0,015	0,022	0,034	1,750	0,015	0,022	0,035	1,764	0,015	0,022	0,035
**Posterior cingulate gyrus**	2,223	0,019	0,022	0,034	2,163	0,018	0,022	0,034	2,280	0,019	0,022	0,035
**Parahippocampal gyrus**	2,636	0,022	0,019	0,029	2,254	0,019	0,020	0,030	2,909	0,025	0,019	0,029
**Insular cortex**	2,445	0,021	0,022	0,031	2,475	0,021	0,022	0,031	2,418	0,021	0,022	0,032
**Anterior insula**	3,048	0,026	0,026	0,037	3,032	0,026	0,026	0,037	3,066	0,026	0,025	0,037
**Posterior insula**	2,859	0,024	0,020	0,032	2,932	0,025	0,020	0,033	2,779	0,024	0,019	0,032
**Central operculum**	2,018	0,017	0,020	0,030	2,044	0,017	0,020	0,030	1,997	0,017	0,019	0,030
**Frontal operculum**	2,001	0,017	0,021	0,033	2,103	0,018	0,021	0,033	1,907	0,016	0,021	0,033
**Parietal operculum**	1,737	0,015	0,017	0,027	1,365	0,012	0,016	0,026	1,995	0,017	0,016	0,028

References

1. Spinnler H, Tognoni G. Standardizzazione e taratura italiana di test neuropsicologici. *Ital J Neurol Sci*. 1987;6 (Supplementum n° 8).

2. Catricalà E, Della Rosa PA, Ginex V, Mussetti Z, Plebani V, Cappa SF. An Italian battery for the assessment of semantic memory disorders. *Neurological Sciences*. 2013;34(6):985-993. https://doi.org/10.1007/s10072-012-1181-z

3. Costa A, Bagoj E, Monaco M, et al. Standardization and normative data obtained in the Italian population for a new verbal fluency instrument, the phonemic/semantic alternate fluency test. *Neurological Sciences*. 2014;35(3):365-372. https://doi.org/10.1007/s10072-013-1520-8

4. Banco E, Veronelli L, Briguglio M, Luzzatti C, Vallar G. The Semantic Association Test (SAT): normative data from healthy Italian participants and a validation study in aphasic patients. *Neurol Sci*. 2023;44(5):1575-1586. https://doi.org/10.1007/S10072-022-06543-5

5. Caffarra P, Vezzadini G, Dieci F, Zonato F, Venneri A. Rey-Osterrieth complex figure: normative values in an Italian population sample. *Neurol Sci*. 2002;22(6):443-447. https://doi.org/10.1007/S100720200003

6. De Renzi E, Motti F, Nichelli P. Imitating gestures: a quantitative approach to ideomotor apraxia. *Arch Neurol*. 1980;37(1):6-10.

7. Mancuso M, Rosadoni S, Capitani D, et al. Italian standardization of the Apples Cancellation Test. *Neurological Sciences*. 2015;36(7):1233-1240. https://doi.org/10.1007/s10072-015-2088-2

8. Schenkenberg T, Bradford DC, Ajax ET. Line bisection and unilateral visual neglect in patients with neurologic impairment. *Neurology*. 1980;30(5):509-509. https://doi.org/10.1212/WNL.30.5.509

9. Aiello EN, Esposito A, Gramegna C, et al. The Frontal Assessment Battery (FAB) and its sub-scales: validation and updated normative data in an Italian population sample. *Neurol Sci*. 2022;43(2):979-984. https://doi.org/10.1007/S10072-021-05392-Y

10. Laiacona M, Inzaghi MG, De Tanti A, Capitani E. Wisconsin card sorting test: a new global score, with Italian norms, and its relationship with the Weigl sorting test. *Neurol Sci*. 2000;21(5):279-291. https://doi.org/10.1007/S100720070065

11. Giovagnoli AR, Del Pesce M, Mascheroni S, Simoncelli M, Laiacona M, Capitani E. Trail making test: normative values from 287 normal adult controls. *Ital J Neurol Sci*. 1996;17(4):305-309. https://doi.org/10.1007/BF01997792

12. Caffarra P, Vezzadini G, Dieci F, Zonato F, Venneri A. A short version of the Stroop test: Normative data in an Italian population sample. *Nuova Rivista Neurologia*. 2002;12:111.

13. Della Sala S, MacPherson SE, Phillips LH, Sacco L, Spinnler H. How many camels are there in Italy? Cognitive estimates standardised on the Italian population. *Neurol Sci*. 2003;24(1):10-15. https://doi.org/10.1007/S100720300015

14. Monaco M, Costa A, Caltagirone C, Carlesimo GA. Forward and backward span for verbal and visuo-spatial data: Standardization and normative data from an Italian adult population. *Neurological Sciences*. 2013;34(5):749-754. https://doi.org/10.1007/s10072-012-1130-x

15. Carlesimo GA, Caltagirone C, Gainotti G, et al. The mental deterioration battery: Normative data, diagnostic reliability and qualitative analyses of cognitive impairment. *Eur Neurol*. 1996;36(6):378-384. https://doi.org/10.1159/000117297

16. Capitani E, Sala S Della, Laiacona M, Marchetti C, Spinnler H. Standardizzazione ed uso clinico di un test di memoria di prosa. *Bollettino di Psicologia Applicata*. 1994;209:47-63.
